# Dosimetric validation of intensity‐modulated bolus electron conformal therapy planning and delivery using an anthropomorphic cylindrical head phantom

**DOI:** 10.1002/acm2.14347

**Published:** 2024-04-04

**Authors:** Andrew S. McGuffey, Garrett M. Pitcher, Kevin J. Erhart, Kenneth R. Hogstrom

**Affiliations:** ^1^ Department of Physics and Astronomy Louisiana State University Baton Rouge Louisiana USA; ^2^ Mary Bird Perkins Cancer Center Baton Rouge Louisiana USA; ^3^ decimal, LLC Sanford Florida USA

**Keywords:** electron intensity modulation, electron radiotherapy, radiation measurement, treatment planning

## Abstract

**Purpose:**

This work investigated the dosimetric accuracy of the intensity‐modulated bolus electron conformal therapy (IM‐BECT) planning and delivery process using the decimal ElectronRT (eRT) treatment planning system.

**Methods:**

An IM‐BECT treatment plan was designed using eRT for a cylindrical, anthropomorphic retromolar trigone phantom. Treatment planning involved specification of beam parameters and design of a variable thickness wax bolus and Passive Radiotherapy Intensity Modulator for Electrons (PRIME) device, which was comprised of 33 tungsten island blocks of discrete diameters from 0.158 to 0.223 cm (Intensity Reduction Factors from 0.937 to 0.875, respectively) inside a 10.1 × 6.7 cm^2^ copper cutout. For comparison of calculation accuracy, a BECT plan was generated by copying the IM‐BECT plan and removing the intensity modulation. For both plans, a 16 MeV electron beam was used with 104.7 cm source‐to‐surface distance to bolus. In‐phantom TLD‐100 measurements (*N* = 47) were compared with both eRT planned dose distributions, which used the pencil beam redefinition algorithm with modifications for passive electron intensity modulation (IM‐PBRA). Dose difference and distance to agreement (DTA) metrics were computed for each measurement point.

**Results:**

Comparison of measured dose distributions with planned dose distributions yielded dose differences (calculated minus measured) characterized by a mean and standard deviation of −0.36% ± 1.64% for the IM‐BECT plan, which was similar to −0.36% ± 1.90% for the BECT plan. All dose measurements were within 5% of the planned dose distribution, with both the BECT and IM‐BECT measurement sets having 46/47 (97.8%) points within 3% or within 3 mm of the respective treatment plans.

**Conclusions:**

It was found that the IM‐BECT treatment plan generated using eRT was sufficiently accurate for clinical use when compared to TLD measurements in a cylindrical, anthropomorphic phantom, and was similarly accurate to the BECT treatment plan in the same phantom.

## 1 INTRODUCTION

Bolus electron conformal therapy (BECT) is a useful and widely available treatment modality indicated for superficial irregular‐shaped planning target volumes (PTVs). Example treatment sites for which BECT is useful include the head and neck^1^, ^2^, ^3^, ^4^, ^5^ and post‐mastectomy chest wall.^6^, ^7^, ^8^ BECT utilizes variable‐thickness bolus to deliver conformal dose to targets with irregularly shaped distal surfaces with variable depths.^9^ The variable thickness bolus is placed on the patient surface to modulate the range of the electrons to conform the therapeutic isodose surface to the distal surface of the PTV.

As summarized by Hogstrom et al.,^10^ accuracy of dose calculation for BECT treatment planning has been investigated for several dose calculation algorithms, including the Hogstrom pencil beam algorithm (PBA),^11^ pencil beam redefinition algorithm (PBRA),^11^ and Eclipse Monte Carlo (eMC).^12^, ^13^ The results of those studies showed that the PBRA was more accurate than the PBA and slightly more accurate than eMC for calculations involving heterogeneities in cylindrical, patient‐like phantoms representing the retromolar trigone and nose anatomies, with the PBRA and eMC both being sufficiently accurate for clinical use.

While BECT is capable of delivering highly conformal dose distributions, in some cases significant hotspots (> 110% of prescription dose) can occur due to gradients in the proximal bolus surface. As shown by Kudchadker et al.,^14^ intensity modulation (fluence reduction) can be used to reduce hotspots and improve dose homogeneity to the PTV while maintaining conformality.

In 2017, Hogstrom et al.^15^ introduced the passive radiotherapy intensity modulator for electrons (PRIME) device, which provides a simple and potentially economical method of delivering intensity‐modulated electron fields. PRIME devices, which are patient‐specific, use tungsten island‐blocks placed in the field to preferentially reduce electron fluence in the region of dosimetric hot‐spots. The benefits of this technique, known as intensity‐modulated BECT, or IM‐BECT, have been described in detail for reducing hotspots caused by bolus gradients, variable source‐to‐surface distance (SSD), and internal heterogeneities.^15^, ^16^, ^17^

Dose calculation for IM‐BECT uses a modified version of the PBRA, referred to as the intensity‐modulated PBRA (IM‐PBRA).^16^, ^17^, ^18^ As described by Chambers et al.^16^ and demonstrated by Hilliard et al.,^17^ initial IM‐PBRA modifications used the PBA to transport pencil beams of negative fluence from each modulator block to the patient surface, reducing the primary electron fluence. Each pencil beam was assumed equal in area to the cross‐sectional area of the modulator island block. Scotto et al.^18^ showed that the accuracy could be improved by implementing measurement‐based empirical corrections to the pencil beams’ fluence to account for electrons scattering into and out of the island blocks.

Currently, the PBRA is only available for clinical use through p.d bolus design software (decimal, LLC, Sanford, FL, USA). The IM‐PBRA with scatter corrections has been implemented in decimal's treatment planning system (TPS), ElectronRT (eRT), which is currently in development and not yet available for clinical use. eRT is capable of treatment planning and PRIME device design for IM‐BECT, as well as design of conformal and uniform bolus, patient‐specific cutouts, and skin collimation.

Demonstration of the accuracy of the IM‐BECT planning and delivery process, which includes the accuracy of the IM‐PBRA dose calculation as implemented in eRT and the accuracy of the manufactured wax bolus and PRIME device, in clinical situations is necessary for clinical implementation. In this work, IM‐PBRA calculated dose for a pair of clinically relevant eRT treatment plans is compared with measurement for a heterogeneous patient‐like phantom and the calculation accuracy is assessed.

## 2 METHODS

### 2.1 Description of tissue‐substitute anthropomorphic phantom

**FIGURE 1:**
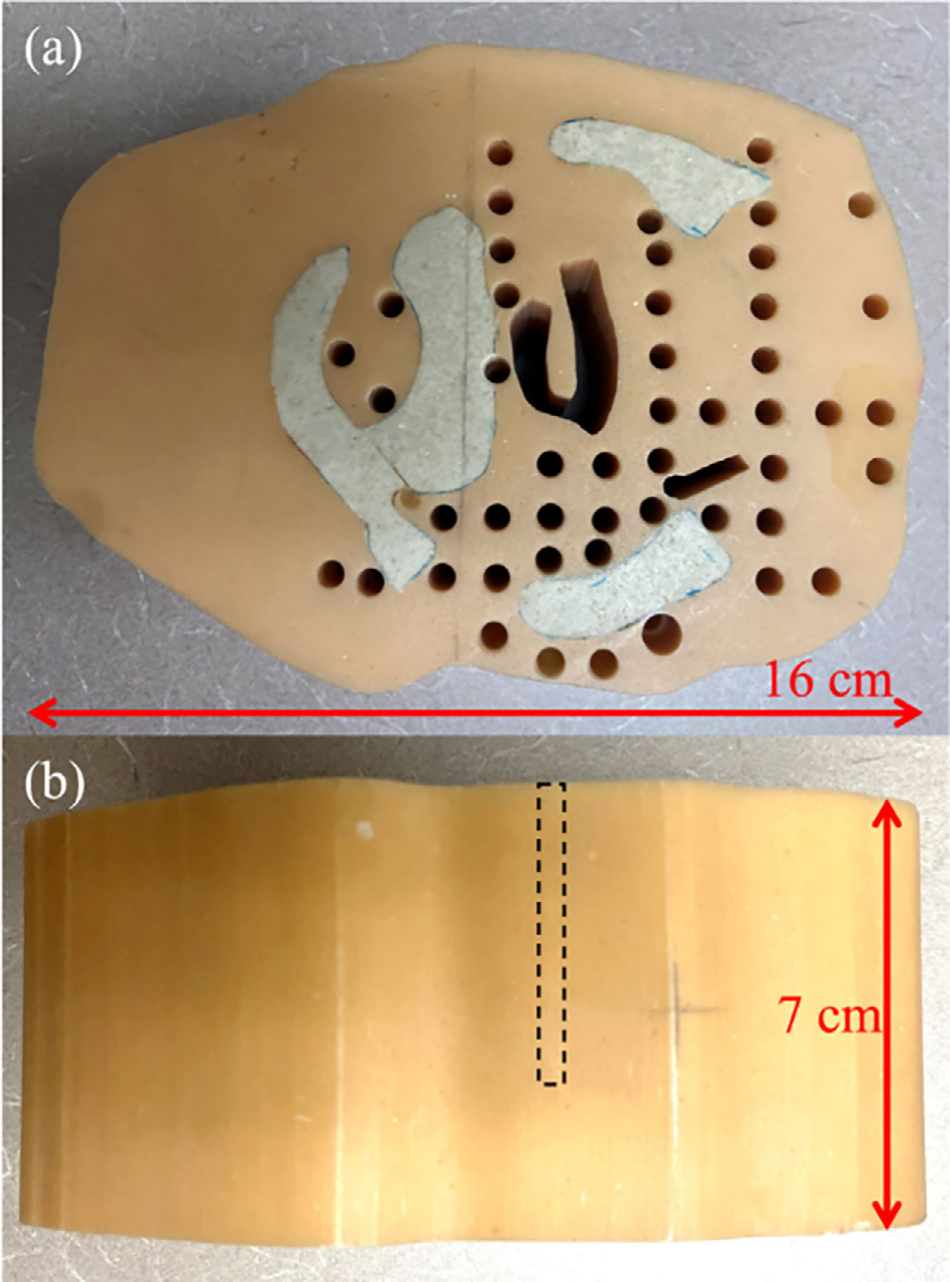
Retromolar trigone phantom, constructed from tissue‐substitute SR4 bone and muscle materials. (a) Top view, showing 47 pre‐drilled, 0.5 cm diameter holes for thermoluminescent dosimeter (TLD) capsules. (b) Beam's eye view, showing the phantom's cylindrical construction. The dashed rectangle illustrates an approximation of the diameter and depth of the drilled holes, which extended approximately 1 cm past the phantom midline.

An anthropomorphic phantom fabricated using a patient's retromolar trigone anatomy was used for this study. The retromolar trigone region was selected as a challenging region for electron dose calculation due to the presence and close proximity of bone and internal air cavities. This phantom was used previously for evaluation of the Hogstrom PBA with two‐dimensional heterogeneity correction^19^ and validation of the PBRA for BECT dose calculation.^11^ The phantom was constructed as a right cylinder based on a single patient CT slice, comprised of SR4 muscle substitute, SR4 bone substitute, and several air cavities, as illustrated in Figure 1.

A detailed description of the composition of the SR4 tissue‐substitute phantom materials is provided in Table 1. Chemical composition^20^ of the phantom materials allowed calculation of collisional mass stopping and scattering powers using NIST ESTAR^21^ and ICRU 35,^22^ respectively. Calculated scattering powers were scaled by (Z + 1)/Z to account for the Møller cross section for electron‐electron scattering.

**TABLE 1:** Composition by weight (%) of SR4 bone and muscle tissue‐substitute materials.

Material	H	C	N	O	Na	Mg	P	S	Cl	K	Ca
SR4 Muscle	9.5	70.25	3.48	15.15	0.08	0.02	0.18	0.50	0.12	0.30	0.01
SR4 Bone	6.4	46.4	2.80	26.4	0.3	0.1	7.0	0.2	0.1	0.2	10.0

*Source*: From Schneider et al.**^20^**

### 2.2 Treatment planning

To initiate treatment planning with the retromolar phantom, CT scans were acquired with a GE Discovery CT590 RT (GE Healthcare, Chicago, IL, USA) scanner with the phantom's cylindrical axis aligned with the CT scanner's bore axis. The scans were acquired with 2.5 mm thickness and 0.443 mm pixel spacing. The CT images were imported into MIM (MIM Software, Beachwood, OH, USA), which was used to transfer the clinical PTV structure, the same PTV used by Carver et al.,^11^ to the new scan. To remove unwanted airgaps from the calculation, the pre‐drilled 0.5 cm diameter holes for the TLD capsules, visible in Figure 1, were overridden as water (HU = 0).

As the SR4 phantom materials are tissue‐substitute, a custom lookup table was used, mapping Hounsfield Units (HU) to their relative stopping and scattering powers (relative to those for water at the same energy), similar to the table reported by Carver et al.^11^ However, because a different CT scanner was utilized for this study, a slightly different table was made to map those values for that scanner.

To determine the stopping and scattering powers of the phantom materials, the process outlined by Carver et al.^11^ was followed. The measured densities of rectangular material samples were used to determine the phantom's CT lookup table for the GE Discovery CT590 RT. The samples were tightly wrapped in layers of flexible tissue‐equivalent bolus (Superflab) to simulate the head geometry and were imaged using the same tube voltage (120 kVp) and scan parameters as the treatment plan images. Measured densities and mean HU for each phantom material are listed in Table 2, along with their relative stopping and scattering powers.

**TABLE 2:** Densities, mean HU, relative stopping power, and relative scattering power of sample materials used to determine CT lookup table.

Material sample	Density (g/cm^3^)	Mean HU	Relative stopping power	Relative scattering power
SR4 Muscle	0.997	−52	0.987	0.851
SR4 Bone	1.628	1245	1.511	2.177
Machinable wax bolus	0.936	−100	0.931	0.731

**FIGURE 2:**
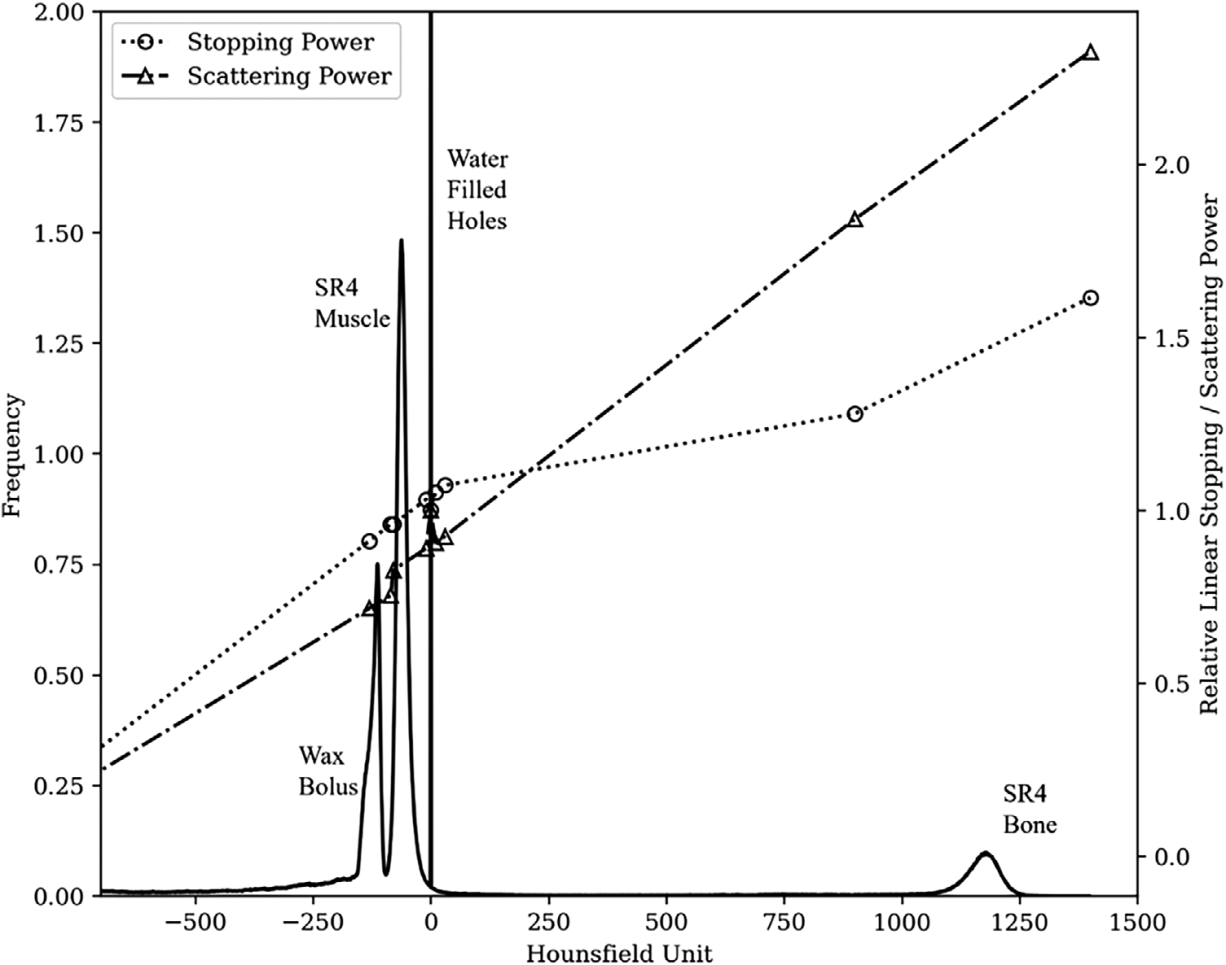
Histogram of HU for the retromolar trigone phantom with bolus. Superimposed are plots of the relative linear stopping and scattering powers from Table 3. HU, Hounsfield Units.

The upper and lower HU bounds for each material were determined by scaling the calculated relative linear stopping and scattering powers by the ratio of the boundary CT number with the mean CT number for each material. The lookup table is presented in Table 3 and plotted in Figure 2.

**TABLE 3:** Lookup table mapping HU to relative linear stopping and scattering powers for the SR4 bone and muscle‐substitute materials and wax bolus.

Hounsfield Units (HU)	Relative stopping power	Relative scattering power	Material
−1000	0.001	0.001	Air
−130	0.912	0.717	Wax bolus
−85	0.959	0.754	
−80	0.959	0.827	SR4 muscle
−10	1.031	0.890	
0	1.000	1.000	Water
10	1.052	0.907	SR4 muscle
30	1.072	0.925	
900	1.279	1.842	SR4 bone
1400	1.615	2.327	

A clinically applicable IM‐BECT treatment plan was developed in eRT (Version 0.9.2; decimal, LLC, Sanford, FL) using the phantom's CT image set. The beam's central axis was aligned with the geometrical center of the PTV, and the lateral extent of the field was defined using a PTV margin of 0.4 cm. Planning was performed using the clinical 16 MeV (*R_90_* = 5.0 cm) beam of the Elekta Infinity at our institution, the minimum available energy sufficient to cover the maximum depth of the PTV (4.5 cm). Dose calculation was performed in eRT using the IM‐PBRA.

The bolus was designed using eRT to conform the 90% isodose line to the distal surface of the PTV using a series of “marching step” operations. This process involves starting with a flat proximal surface and slowly decreasing the bolus thickness, via a series of small steps, only in the areas where the 90% isodose line has not yet reached the depth of the distal PTV surface. Using small steps that only allow the bolus thickness to decrease, results in a stable, convergent bolus design process that achieves ideal dose conformality with minimal hot spots. The dose was normalized such that 100% equaled the given dose, which is the maximum dose along central axis for a beam with the same energy, SSD (to bolus), and rectangular field of minimum area that circumscribes the treatment field.

The PRIME device was designed to reduce hotspots in the PTV caused by bolus shape as well as internal phantom heterogeneities using an IM Dose Scaling factor of 0.98, which controls the extent to which the intensity modulation optimization will modulate the electron fluence to account for cold spots in the dose distribution. A value of 0.98 was selected as a clinically appropriate value and suitable to produce a sufficient number of island blocks with multiple diameters, which was useful for the purpose of validation. Island blocks were positioned in the PRIME device on a hexagonal grid with 0.6 cm packing radius. Block diameters were selected in eRT using the algorithm described by Hilliard et al.,^17^ which selects the diameter that provides the intensity reduction closest to the desired intensity reduction at each grid location. These diameters were selected from the discrete set of diameters available from.decimal.^17^, ^18^

The treatment plan cutout, bolus, and PRIME device designs were sent to.decimal for fabrication. Once fabricated, spot checks were performed on the cutout and bolus to verify that those devices were accurately manufactured. All spot checks were within 0.03 mm for the cutout and 0.08 mm for the bolus, which were within manufacturing tolerance (0.254 mm). All devices were then shipped to our institution for measurement. Once received, a second CT scan was obtained of the phantom with the manufactured bolus using the same scanning parameters and methodology as the initial CT acquisition. The dose distribution re‐calculated on this image set using the IM‐PBRA was used for comparison with measurement.

### 2.3 Description of PRIME device

**FIGURE 3:**
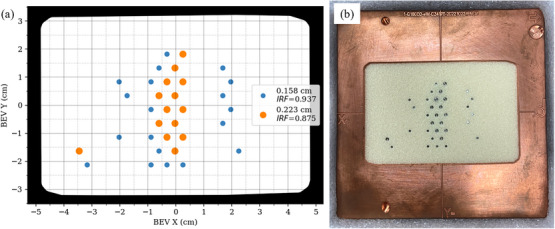
PRIME device for retromolar trigone phantom, with aperture shape and planned island block matrix. (a) BEV diagram of planned device, showing the 33 island blocks, having either 0.158 or 0.223 cm diameter (IRFs of 0.937 and 0.875, respectively). The black region demarcates the shape of the copper cutout. (b) Picture of fabricated device with tungsten cylinders embedded in a low‐density foam plate within the copper cutout, which fits into the 10 x 10 cm^2^ Elekta applicator. BEV, beam's eye view; IRFs, intensity reduction factors; PRIME, passive radiotherapy intensity modulator for electrons.

The PRIME device designed for the retromolar phantom consisted of 33 tungsten island blocks (each 0.6 cm long right cylinders), of either 0.158 or 0.223 cm diameter (defined at block level), having Intensity Reduction Factors (IRFs) of 0.937 and 0.875, respectively, as illustrated in Figure 3. The IRF of an island block is calculated as the local fractional cross‐sectional area of the beam not occupied by the island block, that is, the local fractional area allowing electron transmission through the PRIME device.^15^, ^16^, ^17^, ^18^ Island blocks were positioned on a hexagonal grid with packing radius 0.6 cm. The four distinct columns of island blocks in the device are a result of the cylindrical nature of the phantom. For all calculations and measurements, the phantom's axial dimension was aligned with the *Y*‐axis of the beam's eye view (BEV) in Figure 3. The field shape, approximately rectangular, projected to 10.1 × 6.7 cm^2^ at isocenter.

### 2.4 Factory quality assurance of PRIME device

Factory quality assurance of the manufactured PRIME device was performed following the methodology described by McGuffey et al.^23^ A 52 kVp x‐ray image was used to determine that all island blocks were accurately positioned (maximum positioning error = 0.3 mm), each of correct diameter and having minimal misalignment (maximum *θ* = 2.0°). Differences in intensity maps calculated at a depth of 2 cm in a water phantom (SSD = 103 cm) and 16 MeV with planned (theoretical) and factory‐aligned (measured) island blocks were small, with a maximum intensity difference of 0.5%.

### 2.5 TLD measurements

In‐phantom dose measurements were performed using TLD‐100 dosimetry irradiated with 250 MU, as determined by the eRT treatment plan, with the 16 MeV beam of the Elekta Infinity accelerator at our institution. Following the methodology of Carver et al.,^11^ measurements were performed using cylindrical polyethylene tubes with 5 mm outer diameters and 3 mm inner diameters, which fit snugly inside pre‐drilled holes in the phantom. Approximately 50 mg of TLD‐100 powder (Lot# MDA 10B‐46‐16, Rexon TLD Systems, Inc, Beachwood OH, USA) was inserted into each tube, allowing for three approximately 12.5 mg readings to be acquired per tube.

**FIGURE 4:**
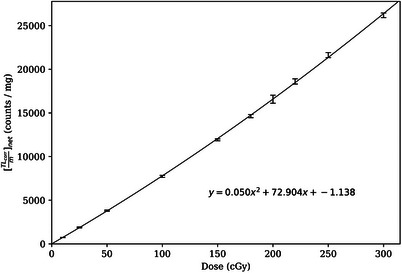
Measured TLD‐100 calibration curve, correlating [*TL_corr_/m*]*_net_* with absorbed dose to water. The error bars represent two times the standard error. The average standard error per net reading (excluding the point with a net reading of zero) was 0.8%. The 2nd‐order polynomial calibration fit is also shown (χ^2^ per degree of freedom = 1.68).

Calibration TLDs were irradiated at 3 cm depth (*R*_100_) in a water‐equivalent plastic phantom along central axis with a 16 MeV beam collimated to 10.53 × 10.53 cm^2^ at isocenter, with 100 cm source to surface distance (SSD). Unlike the phantom TLDs, which were contained in polyethylene tubes, the calibration TLDs were contained in approx. 2 × 2 cm^2^ sealed plastic bags, that is, flat packs. To mitigate effects of airgap around the calibration TLDs, a 0.5 cm‐thick sheet of flexible, tissue‐equivalent bolus (Superflab) was placed immediately below the TLDs. Calibration TLDs were irradiated with 0, 10, 25, 50, 100, 150, 180, 200, 220, 250, and 300 MU. Dose was determined from MU by assuming an output of 1.000 cGy per MU at *R*_100_, which equaled the output specified for our beam model in eRT and facilitated comparisons of measured dose and calculated dose. Dose was correlated with thermoluminescence (TL) signal using a 2nd order polynomial calibration curve as shown in Figure 4.

For each day of irradiations, several (3–4) TLDs were irradiated with 200 MU using the calibration setup. The average reading of those TLDs was used to scale the primary calibration to generate a calibration for each day, accounting for variations in machine output, post‐irradiation readout delay, and daily sensitivity of the TLD readout system.

Phantom irradiations were performed one row of TLDs at a time beginning with most shallow. For each subsequent row, upstream phantom holes were filled with water to remove upstream air gaps that would otherwise perturb downstream electron fluence. Setups for the BECT and IM‐BECT measurements were identical, except for the presence of the PRIME device. The phantom was irradiated to the 250 MU resulting in a delivered given dose of 223.5 cGy.

Phantom positioning was verified prior to each irradiation by comparing room laser positions with marked laser positions at the initial setup each evening and using the optical distance indicator to position the bolus at 104.7 cm SSD, consistent with the eRT treatment plan. Each measured dataset (BECT and IM‐BECT) was repeated once on a different evening to check repeatability, and the average of each TLD measurement was used to compare with calculation. Three TL readings were acquired per TLD measurement for a total of six readings for each measurement position.

TLD readouts were performed 24 to 96 h after irradiation using a Rexon UL‐320 reader (Rexon Corporation, Beachwood, OH, USA). Before any readings were performed, at least five warmup readings were performed with an empty planchette. Approximately 12.5 mg of TLD powder was used for each reading, and three readings were taken for each dosimeter (capsule). The sample mass was measured using a Mettler Toledo AL54 (Mettler Toledo, Columbus, OH, USA) scale. The standard deviation in sample mass was approximately 0.5 mg. However, a preliminary analysis showed no significant correlation between such small variations in sample mass and resultant reading.

Between readings, the planchette was wiped clean of residual TLD powder using a dry cotton swab and weighed to verify that all powder from the previous reading was removed. Routine checks were made to ensure that the temperature curve, which was the measured temperature of the planchette versus time for a single reading and was plotted automatically by the Rexon data acquisition software with each reading, maintained stability throughout a readout session.

For each reading, the light reference counts *LRC_i_*, sample mass *m_i_*, and TL signal (integral PMT counts), *TL_i_*, were recorded. To correct for drift in PMT sensitivity, which was found to decrease approximately 3−4% over a full session of readings (approximately 8 h), each reading's *LRC* (*LRC_i_* corresponding to the *i*th reading for that TLD measurement) was used to determine a correction factor. The corrected, mean *TL* reading for each TLD measurement per unit mass, *TL_corr_/m*, was determined using

(1) $$\frac{TL_{corr}}{m}=\frac{LRC_{nom}}{N}\sum_{i=1}^{N}\frac{TL_{i}}{m_{i}\times LRC_{i}},$$

where *LRC_nom_* is the nominal *LRC*, chosen to be 4000 (an estimate of the convergence value of the *LRC* vs. reading curve), and *N* is the number of reading samples (3) for each TLD.

The net corrected counts per unit mass, [*TL_corr_/m*]*_net_*, were determined by subtracting the background counts per unit mass from the reading counts per unit mass,

(2) $${\left[\frac{TL_{corr}}{m}\right]}_{net}=\frac{TL_{corr}}{m}-{\left[\frac{TL_{corr}}{m}\right]}_{bkgd}.$$

Measured dose was then determined using [*TL_corr_/m*]*_net_* and the calibration curve shown in Figure 4. The standard error in [*TL_corr_/m*]*_net_*, σ_[_ *_TLcorr/m_*_]_ *_net_*, was determined using

(3) $$\sigma_{{\left[\frac{TL_{corr}}{m}\right]}_{net}}=\sqrt{\sigma_{\left[\frac{TL_{corr}}{m}\right]}^{2}+\sigma_{{\left[\frac{TL_{corr}}{m}\right]}_{bkgd}}^{2}}.$$

Measured dose, *D_meas_*, was then determined from [*TL_corr_/m*]*_net_* by inverting the 2nd‐order polynomial fit shown in Figure 4,

(4) $$D_{meas}=\frac{-b+\sqrt{b^{2}-4a\left(c-{\left[\frac{TL_{corr}}{m}\right]}_{net}\right)}}{2a},$$

where *a *= 0.050, *b *= 72.904, and *c *= −1.138. The standard error in measured dose, *σ_Dmeas_*, was then found via propagation of σ_[_ *_TLcorr/m_*_]_ *_net_* through the calibration curve,

(5) $$\sigma_{D_{meas}}={\left[b^{2}-4a\left(c-{\left[\frac{TL_{corr}}{m}\right]}_{net}\right)\right]}^{-1/2}\sigma_{{\left[\frac{TL_{corr}}{m}\right]}_{net}}.$$

This process resulted in two measured values of *D_meas_* and two corresponding standard errors of *σ_Dmeas_* at each measurement position for each treatment plan. The final measured dose at each measurement position for each treatment plan was determined as the weighted average (with weights equal to the reciprocals of the squared uncertainties) of the two dose measurements. The final standard errors were determined using error propagation.

### 2.6 Data processing

For each IM‐PBRA calculation, the DICOM RTPLAN files, containing isocenter and other beam parameters, and RTDOSE files, containing the calculated IM‐PBRA 3D dose distribution, were exported from eRT. The DICOM patient‐based coordinates of the TLD dosimeters in the CT image at the location of TLD powder were determined manually as the centers of the drilled TLD holes in the image, and the calculated values at each measured point were determined using trilinear interpolation of the 3D dose grid. The dose difference (calculated dose minus measured dose) was determined at each measurement point, as was distance to agreement (DTA) for the purposes of comparison. DTA was determined as the minimum distance from the measurement point to the calculated isodose contour of the measured value.

## 3 RESULTS

### 3.1 BECT measurements

**FIGURE 5:**
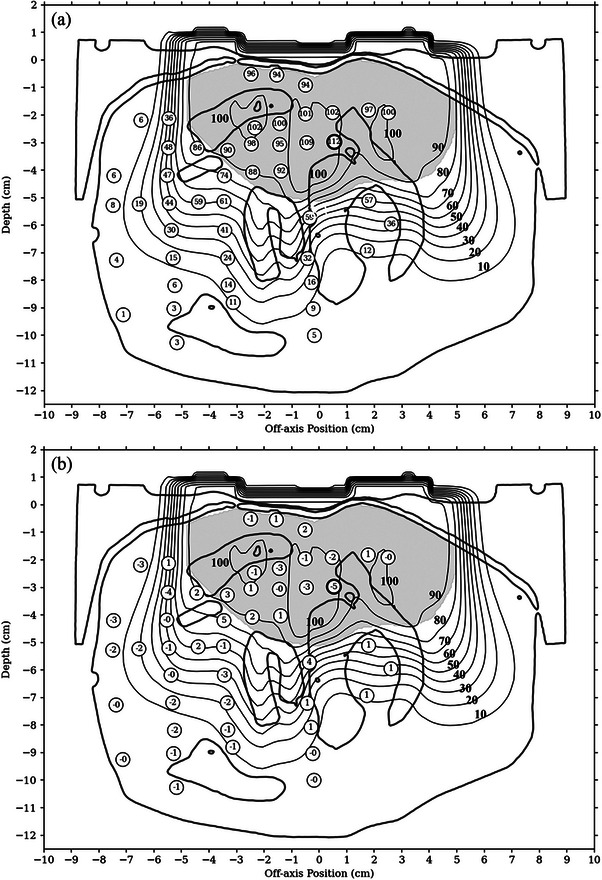
Comparison of measured and PBRA‐calculated dose for the BECT plan in retromolar trigone phantom, with PTV indicated by the shaded region. (a) Points labeled as measured dose. (b) Points labeled as dose difference (calculated dose minus measured). Dose is displayed as a percentage of given dose. Bold circles indicate the absolute valued dose difference was greater than 3% of given dose and that the distance to agreement was greater than 3 mm. BECT, bolus electron conformal therapy; PBRA, passive electron intensity modulation; PTV, planning target volume.

Figure 5a plots isodose curves of the PBRA‐calculated dose (100% = given dose = 223.5 cGy) for the BECT plan with superimposed measured dose points shown in 5a and dose differences in 5b, showing calculated minus measured dose. Measured doses and dose differences are normalized such that the given dose equals 100%. In both, CT image contours delineating material boundaries are plotted. These contours indicated HU values of −150 and 1000. In Figure 5, the off‐axis direction (*x*‐axis) is along the in‐plane direction, with the negative axis pointing toward the gantry. The axes were configured to be consistent with those used to present the results in Carver et al.^11^ The origin is located at central axis on the bolus surface.

**FIGURE 6:**
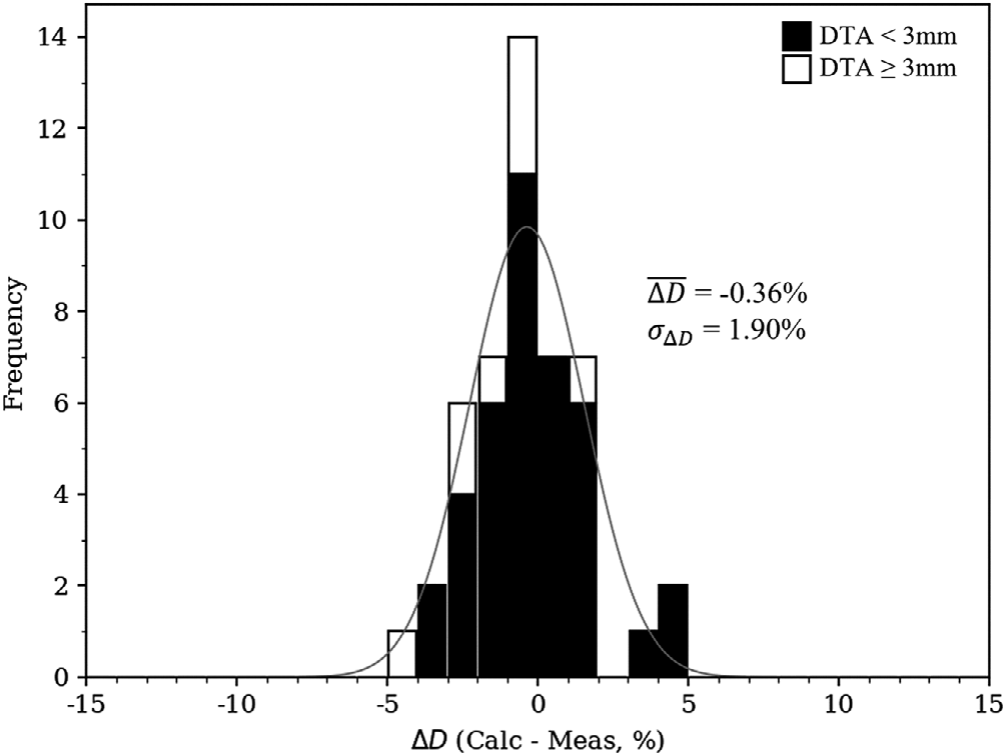
Histogram of dose differences (calculated minus measured) as percent of given dose for the BECT plan. Mean and standard deviation of dose differences are labeled. The parameters of the superimposed Gaussian distribution are the mean and standard deviation of the measured dose differences.

Dose differences plotted in Figure 5b are histogrammed in Figure 6, along with a superimposed Gaussian distribution with parameters equal to the mean and standard deviation of the measured dose differences. The mean and standard deviation dose difference (−0.36% ± 1.90%) are annotated. Measured points with a DTA less than and greater than 3 mm are histogrammed in black and white, respectively. Overall, good agreement was seen between the calculated and measured doses, with a dose difference mean of −0.36% and standard deviation of 1.90%. All dose differences were less than 5%, with 87% (41/47) of measured points within 3%.

**FIGURE 7:**
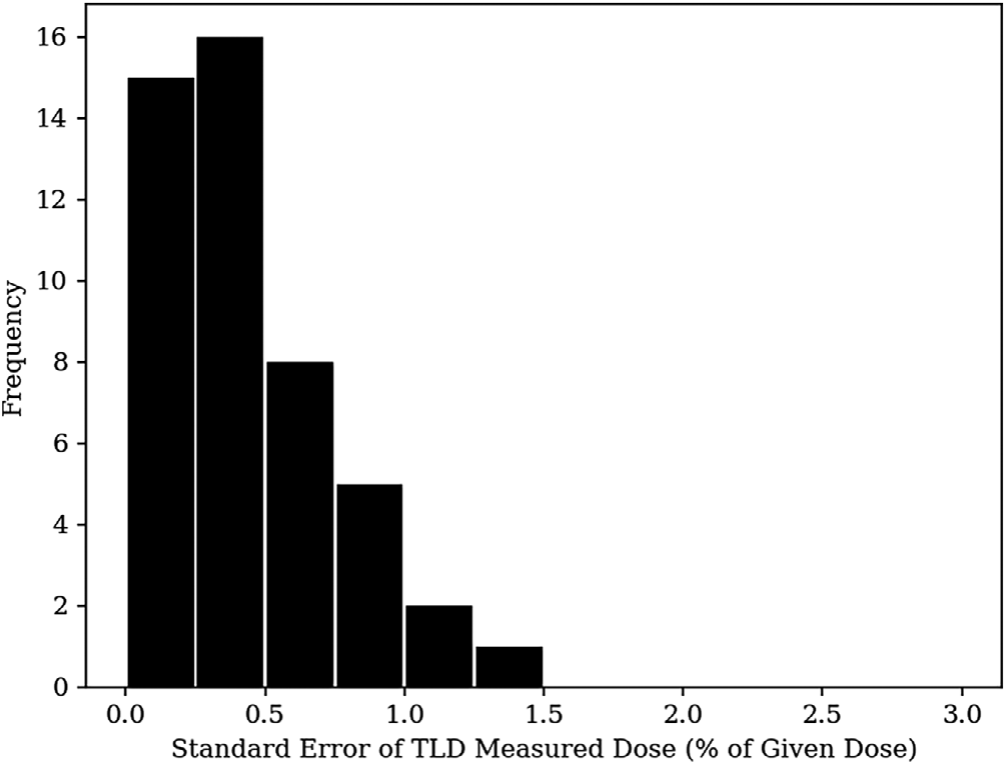
Histogram of standard errors of TLD measured doses for BECT plan. Standard errors are in percent of given dose. The mean standard error for the BECT measurements was determined to be 0.44%. BECT, bolus electron conformal therapy.

Figure 7 plots the binned histogram of standard errors for the BECT TLD measured dose points. Standard errors represent the propagated error due to readout variability, which includes the component due to scaling the daily calibration. The mean standard error of the BECT TLD measurements was calculated to be 0.44%. Root‐mean‐square (RMS) subtraction of the mean standard error resulted in a PBRA standard error of 1.84%.

### 3.2 IM‐BECT measurements

**FIGURE 8:**
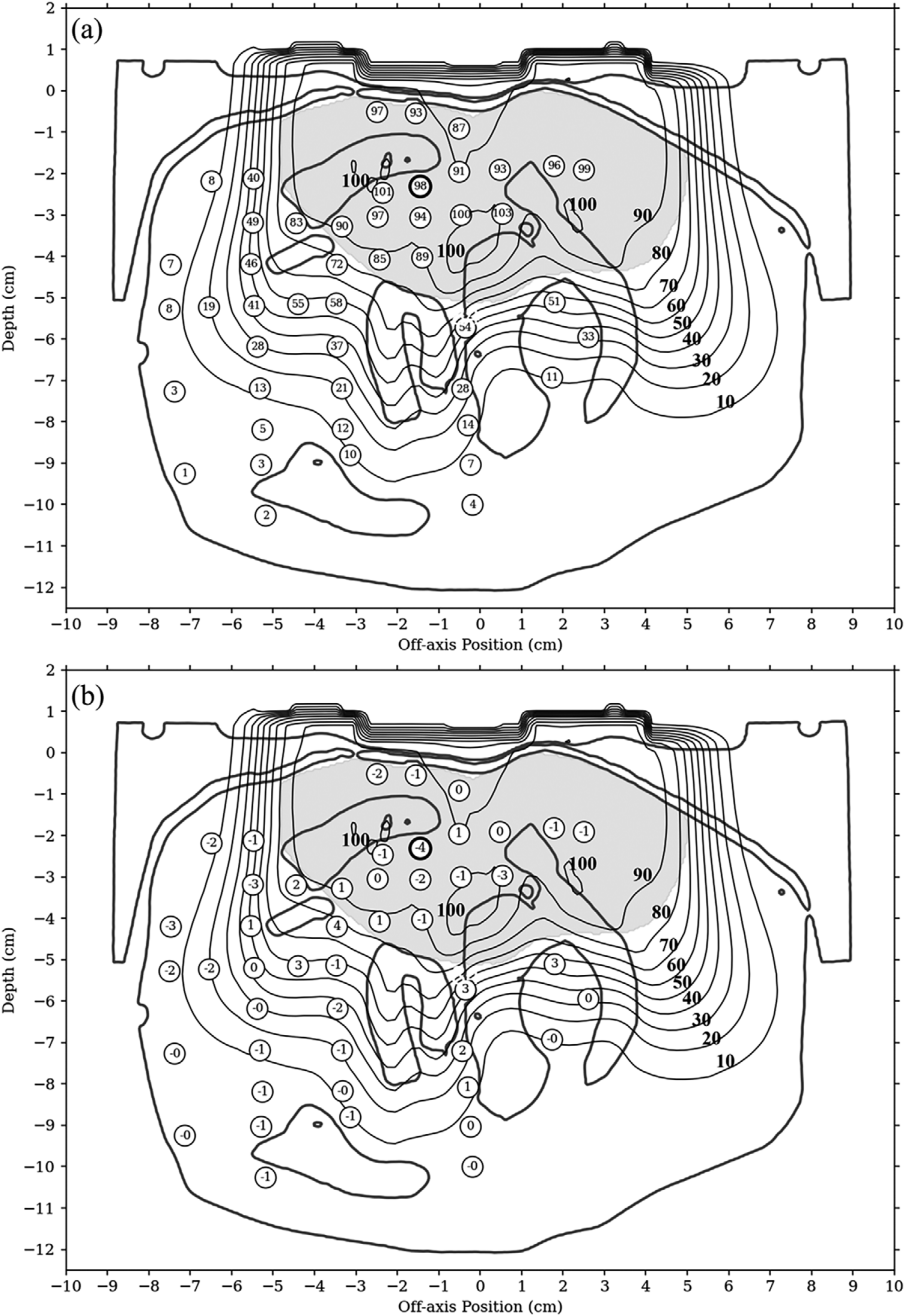
Comparison of measured and IM‐PBRA‐calculated dose for the IM‐BECT plan, with PTV indicated by the shaded region. (a) Circles labeled as measured dose. (b) Circles labeled as dose difference (calculated dose minus measured dose). Dose is displayed as a percentage, normalized such that 100% equals the given dose. Bold circles indicate the absolute valued dose difference was greater than 3% of given dose and that the distance to agreement was greater than 3 mm. IM‐BECT, intensity‐modulated bolus electron conformal therapy; IM‐PBRA, intensity‐modulated passive electron intensity modulation.

Figure 8 compares calculated isodose curves with measured TLD point doses for the retromolar phantom IM‐BECT plan (100% = given dose). Also shown is the outline of the CT volume used for dose calculation. Figure 8a provides measured dose values as a percent of given dose, and Figure 8b provides the dose difference (calculated dose minus measured dose).

**FIGURE 9:**
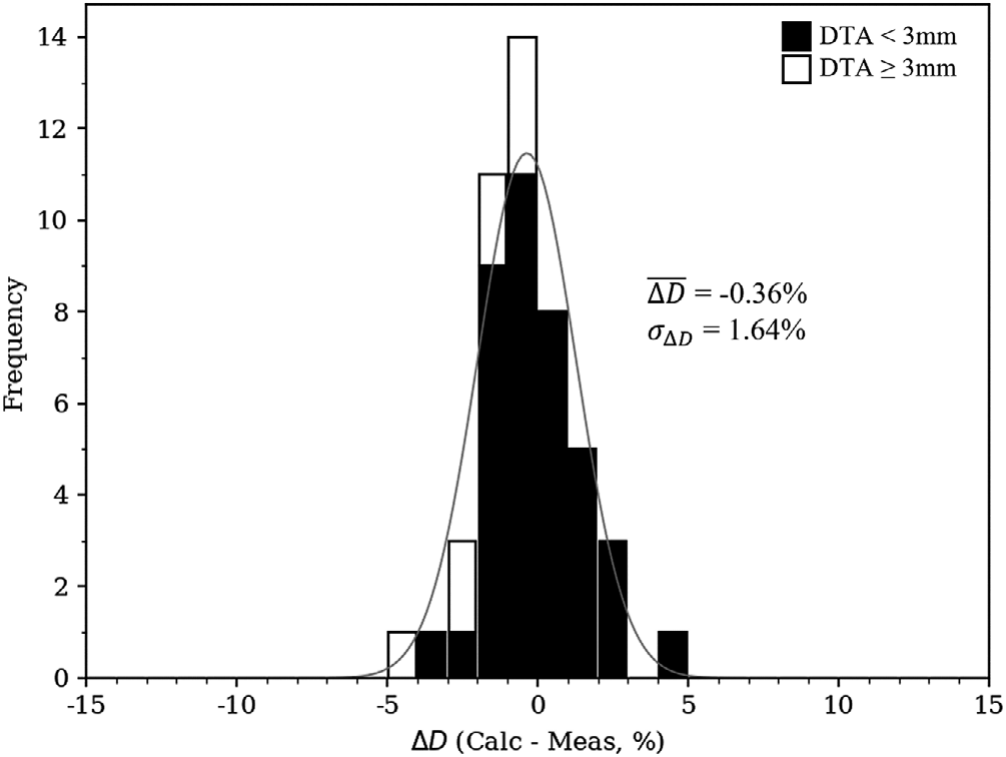
Histogram of dose differences (calculated minus measured) as percent of given dose for the IM‐BECT plan. Mean and standard deviation of dose differences are labeled. The parameters of the superimposed Gaussian distribution are the mean and standard deviation of the measured dose differences. IM‐BECT, intensity‐modulated bolus electron conformal therapy.

Dose differences plotted in Figure 8b are histogrammed in Figure 9, with superimposed Gaussian distribution and annotated mean and standard deviation of the dose difference distribution. The parameters of the Gaussian distribution are the mean and standard deviation of the measured dose differences (−0.36% ± 1.64%). Measured points with a DTA less than and greater than 3 mm are histogrammed in black and white, respectively. Overall, good agreement was seen between the calculated and measured doses, with a dose difference mean of −0.36% and standard deviation 1.64%. All dose differences were less than 5%, with 94% (44/47) of measured points within 3%.

**FIGURE 10:**
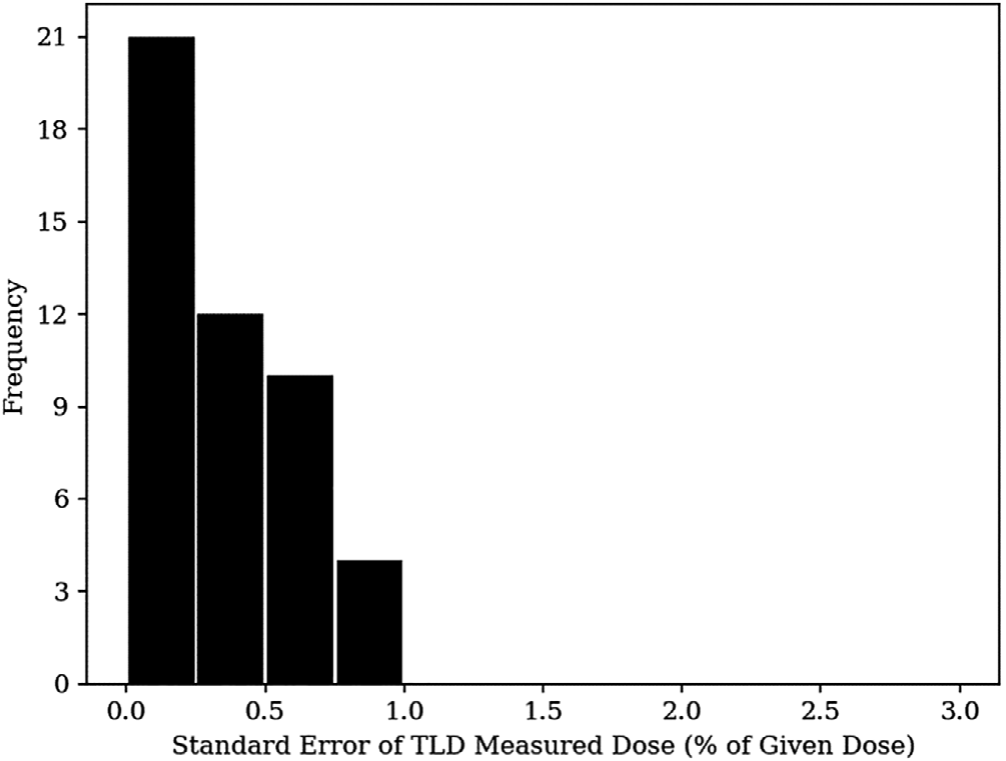
Histogram of standard errors of TLD measured doses for IM‐BECT plan. Standard errors are in percent of given dose. The mean standard error for the BECT measurements was determined to be 0.35%. IM‐BECT, intensity‐modulated bolus electron conformal therapy.

Figure 10 plots the binned histogram of standard errors for the IM‐BECT TLD measured dose points. The mean standard error of the IM‐BECT TLD measurements was calculated to be 0.35 %. RMS subtraction of the mean standard error resulted in a PBRA standard error of 1.60%.

## 4 DISCUSSION AND CONCLUSIONS

This work performed an end‐to‐end test of IM‐BECT planning and delivery, using the IM‐PBRA in.decimal eRT. Results showed that the IM‐PBRA was accurate when compared to TLD measurements in a tissue‐substitute, anthropomorphic phantom for its IM‐BECT plan, and similarly accurate to the IM‐PBRA in the same phantom for the BECT plan. Importantly, a reduction in IM‐PBRA accuracy was not found when intensity modulation was applied, that is, the IM modifications to the PBRA did not result in decreased measured accuracy.

The methods employed in this study follow previous work done by Carver et al.,^11^ which compared PBRA calculation to TLD measurement in the same phantom with and without bolus. Hence, the BECT results presented in this study are directly comparable to those reported by Carver et al., in terms of the expected accuracy and precision of the calculation with respect to measurement. Overall, dose difference statistics are consistent with those determined by Carver et al., with this study reporting BECT dose differences of −0.36% ± 1.90% and Carver et al. having reported −0.20% ± 1.78% in their bolus study with the same phantom. Additionally, these results show similar modest increases in agreement when compared with evaluations of the eMC algorithm of the Eclipse TPS (Varian Medical Systems, Palo Alto, CA, USA),^13^ which reported dose differences of −0.01% ± 2.55% for the same phantom, bolus, and irradiation conditions as presented in Carver et al.^11^

For both BECT and IM‐BECT comparisons, all measurements were within 5% of given dose or within 3 mm DTA of eRT calculation. In both comparisons, 46 out of 47 (97.8%) TLDs were within 3% or 3 mm of calculation. Interestingly, the points not meeting these criteria in the BECT and IM‐BECT datasets were at different locations but were both at a bone‐muscle interface. This suggests that calculational accuracy may be slightly decreased at or near the interface. However, both points had an absolute‐valued difference between calculation and measurement of less than 5%, which is within clinical tolerance for electron TPS validation for inhomogeneities as recommended by Medical Physics Practice Guideline 5.b.^24^

Prior to clinical translation of this technology, standards for patient‐specific quality assurance (PSQA) of IM‐BECT delivery should be established. The use of a diode‐array type device for this purpose, such as the MapCHECK3, has been demonstrated,^25^ and is potentially an easily adoptable solution given the extensive clinical use of these devices for QA of intensity modulated x‐ray therapy (IMXT). Once this technology becomes clinically available, the clinical utility of IM‐BECT should be compared to that of BECT and IMXT for various treatment sites.

## AUTHOR CONTRIBUTIONS

As submitting author, I attest that all coauthors and myself (1) contributed to drafting and/or editing the submitted manuscript, (2) reviewed and approved the final submitted manuscript, and (3) are accountable for the integrity of the material submitted. Each individual contributed in multiple ways to the material reported in this manuscript, and the primary contribution(s) from each author are: Andrew S. McGuffey was a graduate student whose dissertation comprised the bulk of research for this study, particularly treatment planning, TLD measurements, and data analysis. Garrett M. Pitcher supervised the graduate student and contributed to data analysis. Kevin J. Erhart provided the custom wax bolus and PRIME device used in this study, supervised the development of the eRT TPS used in this study, and served as principal investigator on the SBIR grant funding this study. Kenneth R. Hogstrom conceived the concept of intensity‐modulated bolus electron conformal therapy (IM‐BECT), developed the scientific portion of the grant that funded this project, and contributed to data analysis.

## CONFLICT OF INTEREST STATEMENT

Kevin Erhart is an employee of decimal, LLC and principal investigator of NIH Award Number 2R44CA199838‐02 for which Mary Bird Perkins Cancer Center has a subaward. The authors have no other relevant conflicts of interest to disclose.

## Data Availability

The data that support the findings of this study are available from the corresponding author upon reasonable request.
